# COVID-19: a plea to protect the older population

**DOI:** 10.1186/s12939-020-01193-5

**Published:** 2020-05-19

**Authors:** Daniele Carrieri, Fedro Alessandro Peccatori, Giovanni Boniolo

**Affiliations:** 1 College of Medicine and Health & Wellcome Centre for Cultures and Environments of Health, University of Exeter, Exeter, UK; 2 Department of Oncology and Haemato Oncology, University of Milan and European Institute of Oncology IRCCS, Via Ripamonti 435, 20141 Milan, Italy; 3 Department of Biomedical and Specialty Surgical Sciences, University of Ferrara (Ferrara) & Civitas Vitae Research Centre (Padova), Ferrara and Padova, Italy

## Introduction

A recent WHO declaration^1^ reports that over 95% of COVID-19 related deaths in the European Region (currently the epicentre of the pandemic alongside with North America) occurred in people older than 60 years. Elaborating on these figures, we want to make a clear statement to reinforce the protection of each nation’s older adults and to promote intergenerational equity and solidarity. This message deserves to be fully understood in light of the potential discrimination against older people in the current public debate regarding the allocation of scarce medical resources during the present pandemic.

Priority setting in healthcare, i.e. “the task of determining the priority to be assigned to a service, a service development or an individual patient at a given point in time” [1], is unavoidable, because the demand for healthcare exceeds resource availability, particularly in pandemic circumstances. However, even under conditions of resource scarcity, priority setting processes should not compromise the basic human rights to health and wellbeing of any stratum of the population, especially that of older people and other vulnerable groups. Yet, there is a real risk that the immense pressure on resources exerted by the COVID-19 pandemic may result in the prioritisation of key services across different age groups based on actual or future contribution to society, or on other utilitarian criteria.

In this context, older people may be seen as a particularly large and vulnerable group, and one which plays a less active role in society, especially from an economic point of view. Therefore, in an attempt to optimise resources, older people might be considered as having a priori less rights to high-quality healthcare than younger generations, who tend to be seen as less vulnerable and more active members of society. On the contrary, we want to emphasise that resource scarcity and utilitarian calculus should not undermine older people’s rights to receive high-quality healthcare: societies have a moral imperative to protect and respect the dignity and autonomy of older people [2]. Moreover, in line with philosophical accounts which regard ‘vulnerability’ as valuable [3], we also wish to highlight that older people can enrich societies.

We are not claiming that age parameters should not be considered while facing the allocation of scarce sources. Our aim is to strongly oppose the idea of setting an a priori age cut-offs to decide who should get access to healthcare, as such ideas discriminate against older people. We believe that, especially during the COVID-19 pandemic, there is the need to focus on this issue, since there are some initial signs of such discrimination. Thus, we wish to show how these discriminatory phenomena can cause severe negative outcomes not only from the moral standpoint of respect for dignity and autonomy of older people, but also from the utilitarian perspective of achieving the greatest good for the greatest amount of people.

## Initial signs of discrimination of older people?

In Italy – one of the first European countries hit by the pandemic and one among those with a significant proportion of older individuals– SIAARTI^2^ guidelines on admission to intensive care units (ICU) envisage (or at least do not exclude) the possibility of a priori age cut-offs. The main argument is that elderly patients may need longer intubation time and have less chance of survival than younger patients. The argument is also underpinned by the utilitarian calculus of maximisation of benefit for the greatest amount of people [4].

“An *age limit* for the admission to the ICU may ultimately need to be set. The underlying principle would be to save limited resources which may become extremely scarce for those who have a much greater *probability of survival* and life expectancy, in order to *maximize the benefits* for the *largest number* of people. In the worst-case *scenario of complete saturation* of ICU resources, keeping a “first come, first served” criterion would ultimately result in withholding ICU care by limiting ICU admission for any subsequently presenting patient.” (Recommendation 3; *their emphasis*).

This discrimination against older people seems to have spread to other countries which are dealing with the COVID-19 ‘tsunami’. In the US – where there are triage frameworks which do not categorically exclude large groups of patients (therefore also older people) [5], opinion-leader bioethicist A. Caplan, interviewed by a major national newspaper, makes similar age cut-off claims (which are also underpinned by utilitarian calculus):

“Although a patient’s age is not the only consideration, it is one. It would be dishonest if we didn’t say age is a driver. Age is correlated with resilience. Because younger patients, in general, get better faster, they may free up a ventilator more quickly for the next patient”.^3^

Moreover, in the US social distancing measures have been implemented rather late and/or sporadically across different states (including states with populations older than the national average)^4^ effectively putting older people, and other vulnerable groups, at risk. A common argument for such inconsistent implementation was the decision to assign more importance to the economy than to the health of the population – alongside the ‘false myth’ that younger people could not develop serious complications from COVID-19.^5^

## Reviving ideas of a ‘duty to die’?

These initial signs of age discrimination in time of COVID-19 seem to go in parallel with a contemporary dangerous trend. There are, indeed, some scholars supporting the idea both of a priori age-based cut-offs for access to certain kinds of medical intervention, and preaching that older people should sacrifice themselves for the good of younger generations. Before the emergence of COVID-19, an American oncologist and bioethicist, E. Emanuel, sparked some controversy [6] after writing about his plan to self-impose a cut-off of 75 years for access to any ‘curative’ treatment and accepting only ‘palliative’ treatment.^6^ Such proposals echoed the previous 65 year cut-off advocated by D. Callahan [7], and even arguments about the existence of a *duty to die* [8].

The common denominators of these positions include the utilitarian idea that continuing to live after a certain age can impose significant financial burdens to healthcare systems, as well as financial and emotional burdens to family and/or caregivers, younger generations, and society as a whole. Another denominator is the critique of the hype around biomedical innovation and the tendency to ‘overmedicalize death’ which may foster illusions of immortality. Finally, these positions share the idea that living too long can also be a loss for the old persons in terms of their own quality of life.

The same arguments used by those who sustain those a priori age-based cut-offs have previously featured in literature. For example, they are not too dissimilar from the ones used by Trollope in his 1882 satirical dystopian novel ‘The Fixed Period’ [9]. Here he depicts a society that has progressed “beyond the limits of civilisation” and has agreed upon euthanasia (a “decent and comfortable departure”) for the elderly when they reach the age of 67 and a half.

Unfortunately, the abstract and fictional positions sketched above are currently being actualised. For example, in Texas, Lieutenant Governor Dan Patrick, said he would be willing to die to preserve the US economy from COVID-19 public health measures: “as someone who turns 70 next week, he was in the high-risk group, but [ … ] he was willing to give up his life for his six grandchildren”.^7^ In Italy, Father Giuseppe Berardelli, 72, died after choosing to give his respirator to a younger coronavirus patient.^8^

## The importance of protecting older people

An important argument against potential age-related euthanasia or suicide and against the dangerous and immoral stigmatisation of aging, is the fact that older people should have the same rights as others to receive high-quality health care and the rights not to be spurred – especially by key opinion leaders - to consider their own death as something good. Moreover, not all (older) individuals have the same clinical, psychosocial and other contextual features and expectations. That is, what could be good for Emanuel, Callahan, Patrick and Berardelli may not be good for others. A 70-year-old patient may have a better chance of survival and benefit more from intensive or other forms of medical treatment than a 30-year-old one. Notably there are studies showing how the so called “oldest old” – i.e. patients 90 years old or older – with early stage lung cancer can benefit from surgery [10]. Even in the context of the current pandemic, there is revealing news, e.g. in Italy a 101-year-old man^9^ and a 102- year-old woman^10^ have recovered from COVID-19.

These examples are also in line with the WHO ‘Active Aging’ policy framework, that is “the process of optimizing opportunities for health, participation and security in order to enhance quality of life as people age” [11].

Moral imperative of respect for the autonomy and dignity of old people notwithstanding, discriminatory positions are problematic also from a utilitarian and economic point of view. For example, US citizens aged 50 and older represent the 35% of the entire population, account for 40% of the national gross domestic product, and contribute 43% of US taxes [12]. While it is important to honour those who have survived into their later years for their contributions to family and society, it is also important to understand the practical and objective contributions that older adults still make to their communities and their families and, occasionally, even to the world.

It seems that there is a contradictory attitude towards the older population. On the one hand, older people are cajoled, especially for business reasons (the silver economy); on the other hand, as soon as the younger generation is under pressure, older people run the risk of being stigmatised or discriminated [13]. It is also important to highlight that such forms of discrimination can pit one generation against the other, seriously impoverishing the whole fabric of societies.

## Conclusion

Exceptional and austere situations like the current and previous pandemics can elicit the best and the worst of humanity. Despite the current COVID-19 circumstances, or possibly *even more so,* it is key to shape the public sphere in ways that allow to recognise both the vulnerabilities and potentialities of all generations. One way to do so is to respect the individual person, going beyond categories such as age or age groups, and to promote intergenerational equity and solidarity by fostering connectedness across different generations.

Caring and protecting older people is not only about respecting their dignity and autonomy. It opens up invaluable possibilities for all generations to engage with collective memories and traditions – promoting the exchange of skills, knowledge and understanding.

## Data Availability

Materials are not available for this paper.

## Footnotes

1 http://www.euro.who.int/en/health-topics/health-emergencies/coronavirus-covid-19/statements/statement-older-people-are-at-highest-risk-from-covid-19,-but-all-must-act-to-prevent-community-spread

2 http://www.siaarti.it/SiteAssets/News/COVID19%20-%20documenti%20SIAARTI/SIAARTI%20-%20Covid19%20-%20Raccomandazioni%20di%20etica%20clinica.pdf. SIAARTI is the Italian Society of Anesthesia, Analgesia, Resuscitation and Intensive Care.

3 https://www.washingtonpost.com/world/national-security/new-yorks-bioethics-experts-prepare-for-a-wave-of-difficult-decisions/2020/03/28/4501f522-7045-11ea-a3ec-70d7479d83f0_story.html

4 https://www.washingtonpost.com/outlook/2020/04/13/many-parts-america-have-already-decided-sacrifice-elderly/?fbclid=IwAR0eVKnT85Lqed_2UX-354RcIiHKrrangriSd2ERDC-1vpsxF_hUjFLYe3o

5 https://www.weforum.org/agenda/2020/03/coronavirus-young-people-hospitalized-covid-19-chart/

6 https://www.theatlantic.com/magazine/archive/2014/10/why-i-hope-to-die-at-75/379329/

7 https://www.theguardian.com/world/2020/mar/24/older-people-would-rather-die-than-let-covid-19-lockdown-harm-us-economy-texas-official-dan-patrick

8 https://www.bbc.co.uk/news/world-europe-52015969

9 https://ph.news.yahoo.com/extraordinary-recovery-101-old-italian-175333011.html?guce_referrer=aHR0cHM6Ly93d3cuZmFjZWJvb2suY29tLw&guce_referrer_sig=AQAAANCHkM0SyBGHlVZWqNEDarfst6YPYyUiH95o2ZHpbg4HI4S3VDQVhlbfgf9ZNXaX6HrrGSdfJ9Zsa3QhyQcf6r8y8m4HVtUmPpjwrUvL96or9NK5ZzY9keNoJWH6y05iFtOw0JwXmO5ncTIVn3h1ltBg7aO8R7qcEH9AYFbUO1UN&guccounter=1

10 https://edition.cnn.com/world/live-news/coronavirus-outbreak-03-28-20-intl-hnk/h_621a06dfd17af984912e4d1c4d94cbac?fbclid=IwAR1AVOzE3qRsFVT32lC8XHAh3T9GnIZG5nQHfF0Q9GQFLDt6DptOYDnV-vw
